# Fine-scale variation in malaria prevalence across ecological regions in Madagascar: a cross-sectional study

**DOI:** 10.1186/s12889-021-11090-3

**Published:** 2021-05-29

**Authors:** Benjamin L. Rice, Christopher D. Golden, Hervet J. Randriamady, Anjaharinony Andry Ny Aina Rakotomalala, Miadana Arisoa Vonona, Evelin Jean Gasta Anjaranirina, James Hazen, Marcia C. Castro, C. Jessica E. Metcalf, Daniel L. Hartl

**Affiliations:** 1 Department of Organismic and Evolutionary Biology, Harvard University, Cambridge, MA USA; 2 Madagascar Health and Environmental Research (MAHERY), Maroantsetra, Madagascar; 3 Department of Ecology and Evolutionary Biology, Princeton University, Princeton, NJ USA; 4 Department of Nutrition, Harvard TH Chan School of Public Health, Boston, MA USA; 5 Department of Entomology, University of Antananarivo, Antananarivo, Madagascar; 6 Catholic Relief Services (CRS) Madagascar, Antananarivo, Madagascar; 7 Department of Global Health and Population, Harvard TH Chan School of Public Health, Boston, MA USA

**Keywords:** Madagascar, Malaria, Ecology, Spatial variation, Community health

## Abstract

**Background:**

Large-scale variation in ecological parameters across Madagascar is hypothesized to drive varying spatial patterns of malaria infection. However, to date, few studies of parasite prevalence with resolution at finer, sub-regional spatial scales are available. As a result, there is a poor understanding of how Madagascar’s diverse local ecologies link with variation in the distribution of infections at the community and household level. Efforts to preserve Madagascar’s ecological diversity often focus on improving livelihoods in rural communities near remaining forested areas but are limited by a lack of data on their infectious disease burden.

**Methods:**

To investigate spatial variation in malaria prevalence at the sub-regional scale in Madagascar, we sampled 1476 households (7117 total individuals, all ages) from 31 rural communities divided among five ecologically distinct regions. The sampled regions range from tropical rainforest to semi-arid, spiny forest and include communities near protected areas including the Masoala, Makira, and Mikea forests. Malaria prevalence was estimated by rapid diagnostic test (RDT) cross-sectional surveys performed during malaria transmission seasons over 2013–2017.

**Results:**

Indicative of localized hotspots, malaria prevalence varied more than 10-fold between nearby (< 50 km) communities in some cases. Prevalence was highest on average in the west coast region (Morombe district, average community prevalence 29.4%), situated near protected dry deciduous forest habitat. At the household level, communities in southeast Madagascar (Mananjary district) were observed with over 50% of households containing multiple infected individuals at the time of sampling. From simulations accounting for variation in household size and prevalence at the community level, we observed a significant excess of households with multiple infections in rural communities in southwest and southeast Madagascar, suggesting variation in risk within communities.

**Conclusions:**

Our data suggest that the malaria infection burden experienced by rural communities in Madagascar varies greatly at smaller spatial scales (i.e., at the community and household level) and that the southeast and west coast ecological regions warrant further attention from disease control efforts. Conservation and development efforts in these regions may benefit from consideration of the high, and variable, malaria prevalences among communities in these areas.

**Supplementary Information:**

The online version contains supplementary material available at 10.1186/s12889-021-11090-3.

## Background

Malaria remains a major public health concern in Madagascar. There are an estimated more than 2 million cases per year [1] and malaria is still among the top 10 overall causes of death and disability in the country [2]. Concerningly, evidence suggests difficulty in controlling the disease in recent years: per the WHO annual malaria report in 2018 [1], malaria incidence increased substantially in 2017 relative to previous years and Kang et al (2018) [3] estimated the proportion of the population living in high transmission areas (prevalence > 20%) increased more than four-fold since 2011.

In Madagascar, existing malaria data are available from periodic national-level surveys, routine reporting from government clinics, and a small number of field studies. For national surveys, data are usually sparse at the local level and then aggregated into large epidemiological zones (‘faciès épidémiologiques’) that coarsely correspond to areas of the country with different rainfall patterns and elevation [4, 5]. A well-characterized trend is that malaria prevalence is higher on the coasts, and lower in the interior fringe and high plateau. Analysis of clinical reporting data has indicated that the semi-arid south and west are generally more prone to irregular peaks in incidence, while the east is characterized by more regular seasonal cycles [3, 6]. However, clinical reporting data may be a poor indicator of burden in rural areas with poor access to clinical care.

Given that national surveys are under-powered at the local, sub-regional level, and no recent surveys are available for many areas, there is a need for new empirical data on the spatial distribution of parasite prevalence across Madagascar. We analyze a newly generated data set from sampling 1476 households divided among five ecologically distinct regions of the country to explore variation in prevalence (by RDT) within and between rural communities.

A secondary motivation is that high rates of deforestation, largely driven by expansion in the land used for subsistence agriculture by impoverished rural populations [7–9], threatens a high proportion of Madagascar’s endemic biodiversity [10–12]. Several large food security, conservation, and health surveillance projects have launched in Madagascar in recent years prioritizing vulnerable rural communities, including in the northeast, southwest, and west coast regions studied here. As a consequence of continuing deforestation, rural communities are situated in variable landscapes consisting of a mosaic of intact, partially degraded, and cleared forest [7, 13]. Yet, the extent to which these heterogenous landscapes link to heterogeneous patterns in infection remains understudied in Madagascar.

Additionally, rural communities in these forest-periphery areas may face elevated risk as they often have poorer access to health infrastructure and increased exposure to malaria mosquito vectors [14, 15].

In this study, we aim to explore fine-scale spatial variation in malaria prevalence in human communities in Madagascar, and we highlight a subset of the studied communities with relevance for conservation programming. In practice, the observation of spatial variation is relevant for control efforts as it allows prioritizing areas of greatest burden where a small portion of the population contributes a disproportionately large share of infections [16]. These geographic sub-units that contain relatively higher infection rates are sometimes termed hotspots (e.g., [17]) and control efforts may be made more efficient by targeting such hotspots.

## Materials and methods

### Study area

Sampling was performed at 6–7 sites each for five ecological regions in Madagascar. Madagascar features extreme ecological diversity as a result of hypervariability in bioclimatic variables such as rainfall, elevation, and plant communities [18, 19]. The five regions studied here are:

1. the high rainfall northeast (NE) containing humid rainforest, secondary forest and flooded lowland rice paddy agriculture (Maroantsetra district),
2. the slightly lower rainfall and more heavily deforested southeast (SE) (Mananjary district),
3. the more arid south and southwest (SW) containing dry spiny forest-thicket and secondary grasslands (Toliara II district),
4. the seasonally dry west coast (WC) containing dry deciduous forest and secondary grasslands (Morombe district),
5. the high elevation central plateau (CP) containing wooded grassland-bushland mosaic and terraced agriculture (Amoron’i Mania region).

**Fig. 1:**
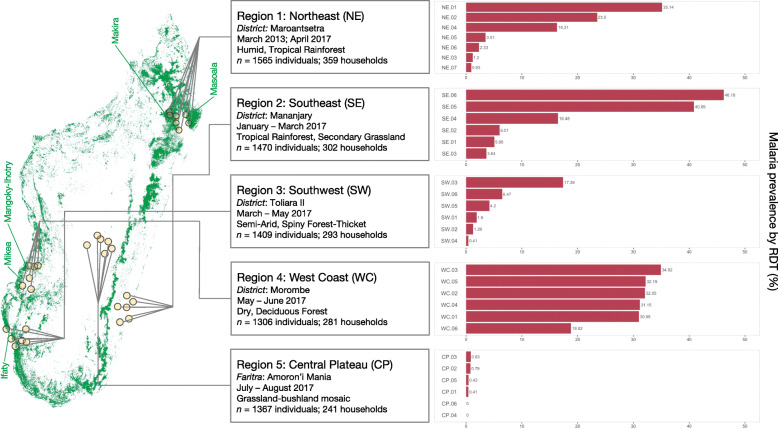
**Sampling in Madagascar and prevalence by ecological region.** Regions as described by predominant vegetation type, their corresponding administrative region (district or *faritra*), sample size, *n*, and the months of sampling (see Supplementary Table 1 for precise dates of sampling and commune names). The map shown is the approximate locations of study sites on the estimated remaining forested areas of Madagascar as of 2014 in green [7, 20], with names of select protected areas adjacent to study communities indicated (see Supplementary Table 1 for full names and distance from study communities to forest edge). To the right, prevalence of malaria infection by rapid diagnostic test (RDT) is shown. Communities are ordered by prevalence within a region. The number of individuals sampled per site ranged from 107 to 284 and the number of individuals positive for malaria ranged from 0 to 127

See Fig. 1 for their locations, names of districts and administrative divisions, sample size, and the time of sampling.

The regions sampled represent a majority of the ecological regions that have been described in Madagascar [21, 22], but do not include two ecological regions: the interior mid-elevation fringe and the sub-desert of the deep south of Madagascar. However, these two unsampled regions are estimated to contribute a small minority of the national malaria burden [4]. Regions were selected to capture the diversity of ecological contexts in Madagascar and to sample within priority zones for large conservation and food security interventions.

Of the five regions sampled, communities in three of the regions reside near (1-15 km) or within buffer zones for protected terrestrial and marine natural areas that are globally acknowledged biodiversity hotspots [23]. NE sites were adjacent to the Makira Natural Park or Masoala National Park, which together contain some of the largest remaining tracts of forest in Madagascar, the last remaining large littoral forests, and a marine reserve. Sites in the SW and WC reside near the Ifaty (sometimes referred to as Ranobe PK32) and the Mangoky-Ihotry and Mikea forests, respectively, where remaining tracts of spiny and dry, deciduous forest have received attention due to being intensely threatened by charcoal production [24]. Three sites in each of the SW and WC regions were also coastal sites adjacent to the Toliara reef system, a 400 + km barrier and fringing reef that is one of the largest in the world. Sites in the SE and CP were farther (20–65 km) from protected areas. See Supplementary Table 1 for approximate distance and full names of protected areas.

Within a region, we hypothesize that (i) communities nearer urban centers have greater access to care and prevention resources, thus motivating the inclusion of some study communities that are more distant, and (ii) that coastal communities may have different vector ecologies due to, for example, sandier soils or winds, and that more mobile fishing communities may have different patterns of exposure. As a result,

rural communities were stratified by two variables in order to attempt to capture the ecological and epidemiological variation present in an area: proximity to the coast and proximity to urban infrastructure (e.g. the nearest town with road access). We defined roads as paths regularly (i.e. daily) passable by public or private vehicles. Lacking census data or maps for these remote areas, communities were defined as sites with clusters of approximately > 30 households visible by satellite imagery. Due to the settlement pattern of rural communities in these areas, household clusters, their peripheral agricultural (mainly rice) fields, and surrounding cleared or deforested lands were easily distinguishable in satellite imagery. Within strata, for each region, communities were randomly selected so as to have communities that were coastal (< 3 km from the coast) or inland (> 3 km from the coast), as well as communities that were within 15 km of an urban center or more than 15 km from an urban center. In the central plateau (CP) region, this inland area is far removed from the coast so by definition there were no coastal communities. The urban centers of the regions studied were Maroantsetra (NE), Mananjary (SE), Toliara (SW), Morombe (WC), and Ambositra (CP). As communities were intentionally stratified to capture variation that may be present within regions, the communities selected are likely not a random sample of variation within a region.

### Sample survey

Details of enrollment, ethical approval, and study design for the larger epidemiological studies from which these malaria prevalence analyses were an extension are explained by Golden et al. 2017 [25], Golden et al. 2019 [26], and Golden and Rice et al. 2020 [27]. Briefly, for the northeast (NE) region, repeated cross-sectional samples, at 3–4 month intervals, were performed between July 2013 – March 2014 for sites NE.01–02 and between August 2016 – April 2017 for sites NE.03–07. To match with the season of sampling in the other four regions (late rainy season), we focus on the March 2014 (for sites NE.01–02) and April 2017 (sites NE.03–07) surveys for the NE. The other regions (SE, SW, WC, CP), were sampled a single time, with the research time sampling each site consecutively between January and August 2017 at approximately the same season (following shortly after a region’s peak rains). The exact date of sampling, and RDT result data, is shown in Additional File 1.

The central plateau (CP) differs notably from the other regions: Malaria transmission is known to be lower [28] and temperatures were colder at the time of sampling due to the austral winter and higher elevation. We expect that prevalence will be much lower in the CP region as a result. Due to not being sampled simultaneously, direct comparison between regions is complicated by inter- and intra-annual fluctuations in malaria transmission. However, sites within a region were sampled within the same seasonal window and on average sites were sampled within 3 weeks of each other.

At each site, approximately 29 households were recruited for enrollment. Households were defined as groups of individuals that regularly cohabited and shared meals containing at least one reproductive aged female and at least one child 5 years of age or younger. Households were randomly selected for recruitment from a census of all households matching these criteria that was updated prior to sampling by consultation with community leaders and heads of family groups. Some communities had less than 29 total households present, in which case all households were approached for enrollment. Household size (the current number of full-time and part-time residents within a household per the head of household) ranged from 2 to 19 (mean 5.2). Latitude and longitude of households were recorded as well as age and sex of individuals.

Individuals of all sexes and ages within an enrolled household were recruited. Comparing reported household size to the number of individuals with a malaria RDT result and matching age and sex data showed that 86% of individuals within the recruited households were sampled for a total of 7117 individuals analyzed here (mean 4.8 individuals sampled per household) **(Fig. S**1). The percentage of individuals within households sampled is similar to that obtained in other studies of household prevalence in Madagascar [30]. Individuals not sampled were either physically absent from the community at the time of sampling, often due to hunting, fishing, or charcoal production activity, or declined to participate in blood sampling. Males aged 15–30 were the most commonly observed to not participate in the blood sampling for those reasons [27].

### Malaria testing

After obtaining informed consent or assent, a blood sample was obtained by veni-puncture or finger prick. Blood sampling procedures were performed by local physicians and phlebotomists – either medical personnel from the local district health office or a trained team member from local partner development organizations with long-standing ties to local communities. Aside from individual consent or assent, efforts to obtain community-wide understanding of the purpose and protocol of blood sampling included public meetings with the community as a whole, with local officials, with traditional community leadership, and with individual family groups (for details, see Golden et al. 2017 [25], Golden et al. 2019 [26], and Golden and Rice et al. 2020 [27]).

Following the manufacturer’s protocol, a portion of the blood sample was transferred to a SD BIOLINE® Pf/Pan (Standard Diagnostics, Gyeonggi-do, Korea, cat. no. 05FK60) rapid diagnostic test (RDT) which uses the antigens HRP2 and pLDH. These RDTs and markers have been assessed in Madagascar previously for the detection of *Plasmodium falciparum* infection [31]. For RDTs with an invalid result, some were repeated if sufficient volume remained from the blood sample after reading the first RDT. Of the 7117 individuals sampled, 963 were positive by RDT (13.5%), and 160 had an invalid RDT result (2.2%) where the control band did not display properly. Individuals testing positive by RDT were offered a consultation with a physician and standard antimalarial medication.

### Analytical strategy

To explore the distribution of infections among households, we performed simulations to compare the observed frequency of multiple infections in a household to that expected under a scenario where infections were distributed randomly. If infections are distributed randomly in a community, the expected number of households with a given number of infections (e.g. the expected number of households with 0, 1, 2, or 3 etc. infections) depends solely on household size (i.e., the number of individuals in the household) and each individual’s independent probability of infection (set equal to the proportion of all individuals that are infected, the community prevalence). A significant excess or rarity of households with multiple infections in comparison to the random expectation would suggest that some variable acting at the household level significantly affects the probability of infection.

We first compared the observed household prevalence to the household prevalence expected in a simulated population with a prevalence set equal to the observed regional average prevalence. The probability of a household having at least one infection is given by 1 – (1 – *P*)^*n*^ where *P* is the probability an individual is infected and *n* is the number of individuals in the household. As a first approximation, we simulated a population where *n* = 4.8 (the mean number of individuals observed per household in our study regions) for each household. All else equal, this provides an estimation for the proportion of households expected to have 1+ infections given the regional prevalence.

We next performed simulations at the community level, using the prevalence of infection at a site and the full observed household size distribution (the number of households of a given size for each site) using 10,000 bootstrap resamples to assess significance. Simulations were performed for each community, using the observed number of individuals in each household sampled in each community. The observed number of infections in a community were randomly re-assigned to households (with the proportion of households of a given size matching the observed distribution in that community). The null hypothesis is that the observed number of households with a given number of infections will be similar to that generated when randomly reassigning infections in a simulated community with a matching distribution of households of a given size. Random reassignment was repeated 1,000 times to determine the probability that an observed distribution of infections among households (for example, as summarized by the number of households with multiple infected individuals) could be expected by chance. Communities where the observed pattern was expected in less than 5% of the permutations were considered to deviate from the null hypothesis.

Analyses were performed in R version 4.0.2 [32]. For the analysis of prevalence by distance, distance between sites was estimated using the latitude and longitude for the mid-point of each study community and the distGeo function in the geosphere R package (version 1.5–10).

### Ethical approval

Details are described in Golden et al. 2017 [25], Golden et al. 2019 [26], and Golden and Rice et al. 2020 [27]. Briefly, approval was obtained from the institutional review board (IRB) committee at the Harvard TH Chan School of Public Health’s Committee on the Use of Human Subjects, the Malagasy Ministry of Health, the ethical committee of the Institut National de Santé Publique et Communitaire in Madagascar, district medical inspectors, and local community leaders (e.g. Président fokontany). Adults provided informed consent for themselves and surrogate consent for infants and children, while other older children provided assent prior to participation. Informed consent was obtained from a parent and/or legal guardian for minors/children below 18 years of age.

## Results

### Variation in prevalence between rural communities in Madagascar

We observed substantial variation in malaria prevalence by RDT between communities within the studied regions (Fig. 1). For the 31 rural sites studied here, prevalence varied from 0% at two sites in the high plateau (sites CP.04 and CP.06, *n* = 247 and 151 individuals) to over 30% at seven sites (site SE.05 and SE.06 in the southeast and sites WC.01, WC.02, WC.03, WC.04, and WC.05 in the west coast region, *n* = 156 to 284 individuals per site) (**Supplementary Table** 1). Prevalence was, as expected, lowest in the higher elevation, cooler central plateau (CP region). The highest observed prevalence was in the southeast sites (Mananjary district), where prevalence was 40.8% at site SE.05 near the Pangalana Canal in Mahatsara Atsimo commune and was 46.2% at site SE.06 in Antsenavolo commune. Despite having two sites with prevalence over 40%, the southeast region also contained three sites with prevalence less than 10% (sites SE.01, SE.02, and SE.03). In the west coast region, among rural communities in Morombe district, prevalence was consistently high and was above 30% in five of the six sampled communities. For comparison, estimated regional average prevalence among children under 5 for the east coast as a whole per the 2016 national MIS was 9.0, and 8.8% for the west coast [4].

**Fig. 2:**
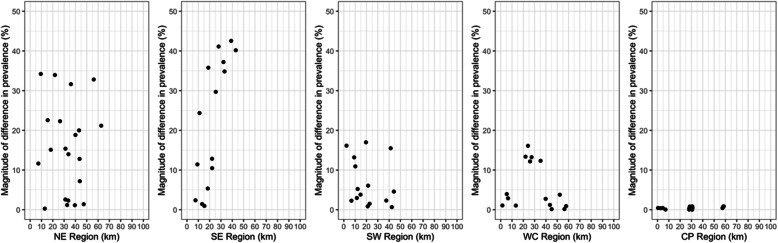
Comparing variation in prevalence between communities versus their geographical distance. Difference in prevalence is shown as the magnitude of the difference between all possible pairwise comparisons between communities. Geographical distance is shown as the distance between the latitude and longitude of the midpoints of communities (in km) (Northeast, NE; Southeast, SE; Southwest, SW; West Coast, WC; Central Plateau, CP)

In several cases, large variation was observed between pairs of geographically close sites (Fig. 2). For example, site SE.01 (prevalence 5.6, 95% CI [2.6–9.7%]) and site SE.05 (prevalence 40.8, 95% CI [35.3–46.6%])) are less than 15 km apart but differed greatly (sampled within 4 weeks of each other). Another example, site SW.02 (prevalence 1.3, 95% CI [0.4–3.6%])) and site SW.03 (prevalence 17.4, 95% CI [13.2–22.5%])) were less than 5 km apart yet also had a large difference in prevalence (sampled within 1 week of each other). Indeed, the variation in malaria prevalence seen between sites within 15 km was often greater than that seen between distant sites. These differences cannot be explained by differences in age between the sampled individuals as the magnitude of difference was similar when constraining comparison to only between the children aged 0–15 sampled in these communities (SE.01: 4.3% [1.4–12.0%] vs SE.05: 33.8% [45.1–60.4%]; SW.02: 1.3% [0.4–4.7%] vs SW.03: 19.2% [13.8–26.1%]).

Atsimo Andrefana, a large administrative region in western and southwestern Madagascar, is worthy of mention as it contains areas within both the semi-arid spiny forest southwest (SW) ecological region (sampled in the Toliara II district) and the dry, deciduous forest west coast (WC) ecological region (sampled in the Morombe district). The pattern of consistently high prevalence seen among the rural sites in the Morombe district (WC region) near Mikea National Park and the Mangoky-Ihotry Protected Harmonious Landscape differs greatly from that seen in the Toliara II district (SW region) near Ifaty forest (Fig. 1) indicating caution is warranted when aggregating data for this administrative unit.

As sampling was not performed simultaneously at the study communities within a region, the length of time between sampling dates may contribute to the variation seen between communities. We observed that communities sampled within 1–2 weeks often differed as much as the sites sampled more than 2, 4 weeks apart, indicating time difference is unlikely to explain the difference in prevalence observed between communities (**Fig. S**2).

### Variation within communities: age and sex

**Fig. 3:**
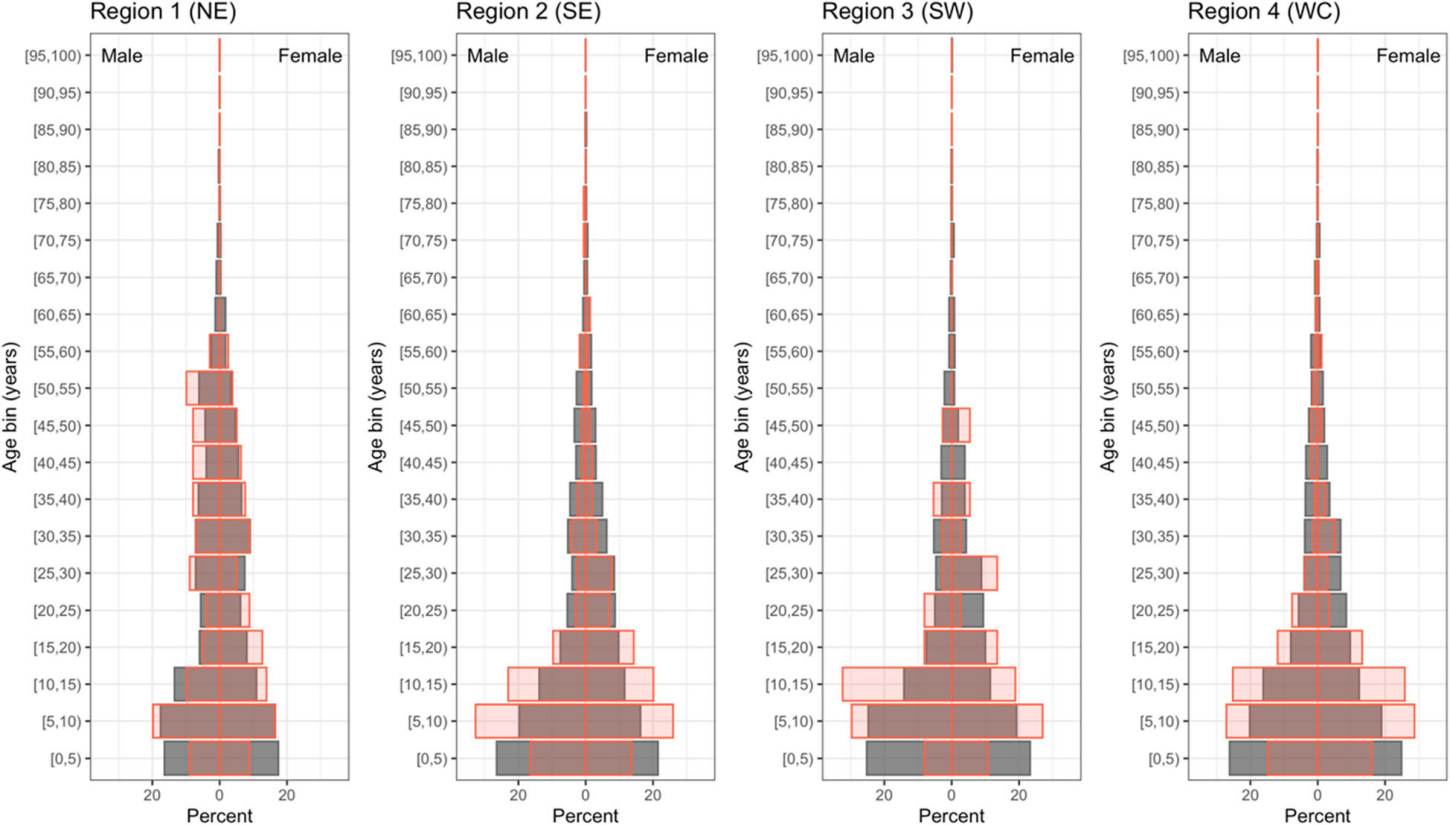
**Age and sex distribution of infections by region.** The distributions of all individuals (gray) and infected individuals (red) are shown by region (Northeast, NE; Southeast, SE; Southwest, SW; West Coast, WC). The proportion of individuals within a 5-year age bin is shown with males on the left and females on the right. Age structure data not shown for the Central Plateau (CP) as only 6 total positive individuals were observed in the region

To explore variation in the distribution of infections within the rural communities in Madagascar, we first characterized the age and sex distribution of infections. For age, a higher proportion of infections were seen among individuals 5 to 20 years of age than other age groups (see Fig. 3). Prevalence among children aged 2–10 was generally higher than the community-wide prevalence (**Supplementary Table** 1). In communities in the southwest, boys aged 10–15 contributed more infections than any other age-sex class. This differs from the pattern typically seen in endemic settings in sub-Saharan Africa where infections are generally most common in younger children and decrease quickly after age 5 [34]. Also of note is that previous analyses (e.g., [4, 35]) of prevalence in Madagascar have focused on the 0–5 year age group and as a result may underestimate population-wide prevalence. The age and sex distribution of infections in the central plateau (CP) region communities sampled could not be determined due to the few RDT positive individuals (*n* = 6) observed. For sex, there were small differences in the percent of infections that were male or female among some age groups in some regions, but no consistent pattern was apparent. Overall, the percentage of sampled individuals that were male (46.3%) was similar but slightly less than those that were female (36.7%), whereas the percentage of infected individuals that were male (36.5%) slightly exceeded those that were female (46.5%).

### Variation within communities: distribution of infections among households

**Fig. 4:**
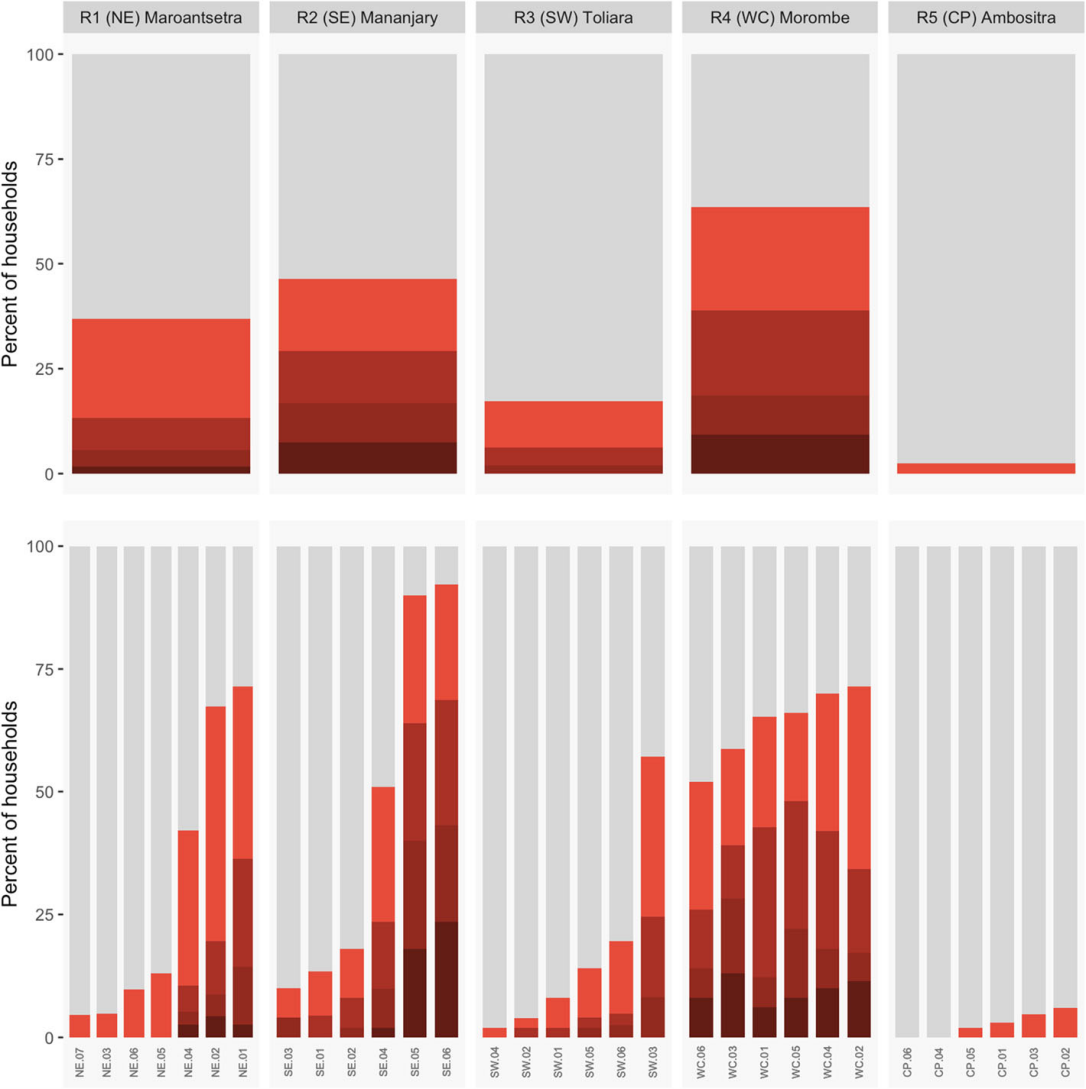
**Households by the number of infected individuals in the household.** The percent of households with a given number of infected individuals (positive by RDT) is shown with darker shades of red corresponding to higher counts of infected individuals. Note that no RDT positive individuals were observed at sites CP.04 and CP.06

In order to explore the distribution of infections among households within a community, we determined the proportions of households found with 0, 1, 2, 3, or 4+ individuals testing positive for malaria by RDT by region (Fig. 4). In many communities in the SE, SW, and WC regions, a high proportion of the households sampled contained at least one individual infected at the time of sampling. This varied from 17.2% of households containing a malaria infection in the SW (Toliara II district, southwest) to 63.6% of households containing a malaria infection in the WC (Morombe district, west coast) (**Supplementary Table 1**). Alarmingly, a substantial proportion of households in the SE (Mananjary district, southeast, 7.4%) and the WC (Morombe district, west coast, 9.3%) were burdened with 4 or more infections at the time of sampling.

Observing a substantial proportion of households with multiple infections has implications for control efforts and indicates burden may be disproportionately high for some households, even in regions with moderate overall average prevalence. For example, 8 of 8 individuals in a household at site SE.05 were positive for malaria by RDT at the time of sampling.

**Fig. 5:**
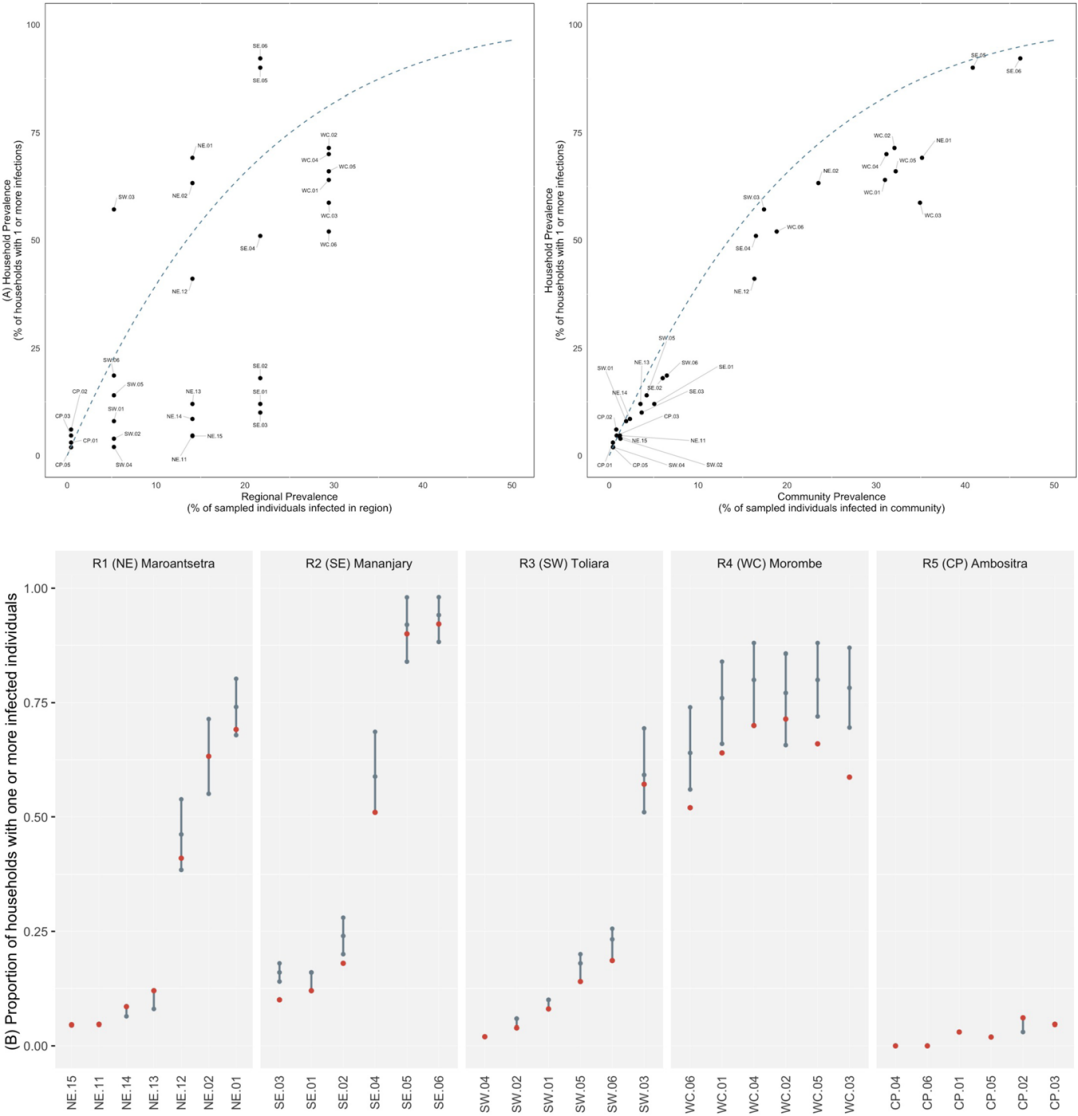
**Distribution of infections among households. A.** Regional prevalence and community prevalence vs household level prevalence (the percentage of households in a community with malaria infections). Dashed blue line is the expected household prevalence from the random distribution of infections among households with a mean of 4.8 individuals per household (see methods). **B.** Comparing the observed number of households with one or more infections for a community (shown with red dots) to the distribution expected from simulations using observed household structure and the observed community prevalence. 95% confidence intervals, as determined by permutation (10,000 replicates), and the median are shown in gray. Within regions, communities are ranked by prevalence. Communities with significant deviations from the null expectation obtained from simulations are shown with the red dot outside of the gray bars representing the 95% confidence intervals of expected values if infections were randomly distributed

However, to determine whether the observed level of household clustering of infections was significantly different from that expected by randomness alone, we performed simulations where infected individuals were randomly re-assigned to households. Such permutation tests allow comparing the observed number of households without infections to the number of households expected given the distribution of household sizes and local prevalence. An excess of households with multiple infections requires that a small number of households in a community contain a disproportionately high number of infections. For many sites, we observed more households with multiple infections than expected from a region’s average prevalence (Fig. 5A).

Next, we tested if the observed frequency of households with a given number of infections could be predicted from community level, as opposed to regional level, prevalence. Indicating infections are clustering non-randomly at the household level in some sites, 6 of the 24 communities analyzed had counts of households with multiple infections that significantly deviated from the range expected from simulations accounting for the proportion of the community that was infected (Fig. 5B). These six communities consisted of four in the WC region and two in the SE region. However, for the majority of communities, the distribution of households with a given number of infected individuals observed in our sample could be predicted from the community level prevalence.

## Discussion

### Malaria as a priority in forest-periphery sites in Madagascar

Rural communities in Madagascar have received attention from a public health perspective due to rates of malnutrition [37–39], anemia [40–42], and poverty [43–47] that are among the highest globally. In addition, high rates of ecological disturbance such as deforestation have led to recent efforts to combine development, conservation, and/or health activities in communities near biodiversity priority areas (e.g, Blue Ventures Community Health, Madagascar PHE Network, WWF Sustaining Livelihoods, Conservation International Sustainable Landscapes in Eastern Madagascar, WCS Health and Education, [25, 48, 49]). Our data suggest malaria prevalence is variable and, in many communities, high in such areas and indicates programming aimed at nutrition, poverty, and habitat preservation may benefit from increased attention to malaria interventions. Warranting particular attention, prevalence was unexpectedly high among communities in the west coast region (Morombe district) situated within the Mikea and Mangoky-Ihotry forested areas (see comparison to model-based estimates from other studies below).

Recent progress in the routine reporting of malaria cases by government health facilities has improved our understanding of malaria infection rates across Madagascar [35, 50, 51]. However, there are several well-documented limitations to case reporting data, including that only a small percentage of infected individuals seek care at a health facility in Madagascar [47, 52] and that rural health facilities report only a minority of their RDT results [50].

Approaches that combine empirical prevalence data, such as that generated in this study, and other passively collected data are a potential way forward in the face of the limitations to case reporting. Arambepola et al (2020) [35], for example, combined prevalence data from the national 2016 malaria indicator survey (MIS) with monthly reported case data from health facilities. At a regional level, our results are consistent with the mapping derived from the model-based predictions of Arambepola et al, as the southeast and western coastal regions were observed to have the highest prevalence in both our empirical study and their model-based estimates. However, our observations of prevalence greater than 30% in some west coast communities sampled near the Mikea forest in 2017 exceed the model-based predictions for the area from Arambepola et al, and notably, we saw large variation between nearby communities within the zones of elevated prevalence identified by their model.

### Evidence of local hotspots in rural Madagascar

In terms of household prevalence, observing high burdens at the household level (e.g., household prevalence > 29% in 10 of 31 communities, Fig. 4) is perhaps unexpected in regions of Madagascar, that from previous estimates, have modest regional average prevalence (e.g., below 10%). This indicates that aggregated, regional prevalence is a poor predictor of burden at the household level (Fig. 5A) and that factors acting at finer spatial scales (i.e., at the community or household level) drive variation in malaria burden in rural communities in Madagascar. An important limitation is that interpretation of household prevalence observations is susceptible to random error due to the limited size of households (e.g., 4–5 individuals) and the number of households sampled (here, *n* = 22–85 per site). Illustrating this, even for sites where there was wide variation in the proportion of individuals infected in a household, the household distribution of infections observed could be produced by random chance (Fig. 5B). For example, at site SE.05, the level of household clustering was not significant but 24 of 29 households (46%) had half or more of the individual members of the household test positive. This illustrates the perhaps counter-intuitive result of the simulations that due to variation in prevalence between sites, variation in household size, and randomness, some households can be expected to have many infected individuals at a time even in the absence of a deterministic process driving clustering or high local prevalence. The implication is that high malaria burden within some households in Madagascar, which likely has broad negative effects on household wellbeing, can be expected even in regions with low average prevalence due to community and household level effects.

In the west coast region, four of six communities had evidence of significant household level clustering and in the southeast, two of six communities were observed with significant clustering. Interestingly, the two sites in the SE (SE.02 and SE.03) with household clustering had lower prevalence than a majority of the other sites sampled in the region such that a small number of households were responsible for a large fraction of infections. For site SE.02 for example, just 8% of the households contributed over 60% of the RDT positive results observed. Policy implications are clear – if the factor(s) responsible for such an uneven distribution of infection can be identified then interventions targeted to vulnerable households can be deployed.

Multiple factors may drive the observed variation in prevalence at the household and community level, including variation in the abundance of mosquito vector larval sources, variation in exposure to bites of adult mosquito vectors, and variation in access to prevention and care. However, the observation of communities and households with elevated prevalence, termed hotspots, are potentially confounded by several factors. Foremost, temporal fluctuations in infection rates, while common in malaria, cannot be inferred from cross-sectional sampling [35]. As a result, it is possible our observations of high prevalence at some sites could reflect short-term or random fluctuations and not be indicative of underlying spatial variation in the risk of malaria infection.

However, multiple lines of evidence suggest that the large variation observed between sites within a region is not an artefact of sampling alone. First, nearby sites that were sampled within 1–2 weeks of each other (equivalent to a single generation in the parasite life cycle) were observed to vary greatly (Fig. 1, **Fig. S**2). Second, sampling at the regions with the highest observed prevalence occurred after (albeit shortly after) the typical seasonal peaks in incidence [3, 51] (see **Supplementary Table** 1) and the time periods of sampling were not atypical as inferred by clinical reporting data [51]. This would suggest that prevalence at the sites sampled was not affected by unusual weather events and may have reached even higher levels prior to the time of sampling. An exception is the central plateau region (CP) where sampling occurred during colder months (July–August) where local transmission is limited. Third, if site-site variation was explained by random fluctuations arising from the sampling strategy, then we would expect a similar magnitude of variation in all regions. However, prevalence at sites in the west coast (WC) was determined with the same sampling strategy but was much less variable than in other regions (Fig. 1). Fourth, if the high prevalence observations were a result of false positive RDT results then we would expect uniformly high prevalence results when applying the same methods across sites. As expected, prevalence was observed to be much lower in the higher elevation CP region sampled at the coldest season of the year, making it unlikely that systematically biased RDT results explain the observed pattern.

One possible hypothesis that could explain the observed local variation in prevalence is that the variation observed between nearby locales is a consequence of rapidly increasing and decreasing local outbreaks. Analysis of genetic data can be of use to test this hypothesis as sporadic, temporary outbreaks of malaria, as opposed to stable local transmission, are expected to result in different patterns of genetic variation [31]. Recent analysis of clinical reporting trends indicate that temporal instability is greater in the more arid southwest and west coast while seasonal cycling is more regular on the east coast [3], which counters our observation of large site-site variation in both the SE and SW, but not the WC regions. If variation over small spatial scales is a consequence of temporary increases in local transmission, then we would expect fewer unique parasite genotypes to be circulating at a given site. Repeated bottlenecking in the local parasite population as transmission rises and falls rapidly over time would reduce the number of distinct genotypes that could arise via recombination. Conversely, less site-site variation in the WC may correlate with more local stability in malaria transmission over time at a given site, in which case more opportunities for recombination among parasite lineages would produce a higher frequency of novel genotypes. If hotspots are ephemeral, then interventions should focus on rapid response, while stable, entrenched reservoirs of high transmission indicate that interventions should focus on extending coverage to remaining unresolved hotspots.

Further investment in active case detection, augmented by genetic analysis, in these areas is a potential path towards understanding the relationship between spatial variation and temporal variation at the local scale. Such data on the location, and stability, of local hotspots is urgently needed to inform control efforts in Madagascar. Regarding future active surveillance efforts, our data suggest expanding the age group of focus to include all children (rather than the 0–5 age group exclusively). RDT positivity among older children was consistently higher than for younger children (Fig. 3), indicating estimates derived only from children of 0–5 years may underestimate community-wide prevalence. Using prevalence among children of 2–10 years has been proposed as a standard for comparison [29] and our data suggest it is likely to better capture overall prevalence. Additionally, the observation that prevalence remained high among children of 10–15 years indicates further study of the pathways of exposure to infection for this age group, which may differ from younger children.

In comparison to other observations of fine-scale variation in malaria infection, hotspots of stable high transmission down to the single household (i.e., homestead) level were observed in coastal Kenya [33, 53] and high levels of spatial heterogeneity have been observed across transmission contexts (for e.g., in Angola [36] and reviewed in [54]). Prioritizing such hotspots to combat malaria transmission has been considered as a result. A recent review of the impacts of spatially targeted interventions, however, argues that it remains difficult to demonstrate targeting hotspots can expedite progress towards malaria control [54]. This indicates, that while data on local hotspots, such as that generated in this study, can give governmental and non-governmental actors areas to target, plans for future interventions need to incorporate rigorous, ongoing assessment.

## Conclusion

From sampling 1476 households across multiple regions of Madagascar, we provide new insight into the heterogeneous landscape of malaria infection in rural Madagascar. Large site-site variation in communities in some areas and evidence of a nonrandom distribution of infections among households indicate the presence of local foci of elevated malaria risk. Such local hotspots merit increased control efforts that are likely to intersect with ongoing in situ conservation and development programming priorities.

## Supplementary Information

**Additional file 1: **csv contains the malaria rapid diagnostic test outcome data as a line list. **Supplementary Table S1**.csv contains sampling dates and malaria prevalence data by site and household. **Figure S1**. shows the distribution of household size (number of individuals per household). **Figure S2**. shows the difference in prevalence as a function of the length of time between sampling dates All files and the R code used to process the data for this study are also publicly available at the GitHub repository: https://github.com/labmetcalf/madagascar_malaria_prevalence_2021.

## Data Availability

The data supporting the conclusions of this article are included within the article and its additional files. Additional File 1 contains the malaria rapid diagnostic test outcome data. Supplementary Table 1 contains malaria prevalence data by site and household. All files and the R code used to process the data for this study are also publicly available at the GitHub repository: https://github.com/labmetcalf/madagascar_malaria_prevalence_2021

## Abbreviations

RDT: Rapid diagnostic test
NE: Northeast Madagascar (Maroantsetra district)
SE: Southeast Madagascar (Mananjary district)
SW: Southwest Madagascar (Toliara II district)
WC: West coast Madagascar (Morombe district)
CP: Central plateau Madagascar (Amoron’i Mania region)
IRB: Institutional review board
MIS: Malaria indicator survey

## References

[1] WHO | World malaria report 2018. 2019. https://www.who.int/malaria/publications/world-malaria-report-2018/en/. Accessed 26 Jan 2021.

[2] Vos T, Lim SS, Abbafati C, Abbas KM, Abbasi M, Abbasifard M (2020). Global burden of 369 diseases and injuries in 204 countries and territories, 1990–2019: a systematic analysis for the Global Burden of Disease Study 2019. Lancet..

[3] Kang SY, Battle KE, Gibson HS, Ratsimbasoa A, Randrianarivelojosia M, Ramboarina S (2018). Spatio-temporal mapping of Madagascar’s Malaria Indicator Survey results to assess *Plasmodium falciparum* endemicity trends between 2011 and 2016. BMC Med..

[4] Institut National de la Statistique (INSTAT), Programme National de lutte contre le Paludisme (PNLP), Institut Pasteur de Madagascar (IPM) et ICF International. 2016. Enquête sur les Indicateurs du Paludisme 2016. Calverton, MD, USA : INSTAT, PNLP, IPM et ICF International.

[5] Mouchet J, Blanchy S (1995). Particularities and stratification of malaria in Madagascar. Sante..

[6] Ihantamalala FA, Rakotoarimanana FMJ, Ramiadantsoa T, Rakotondramanga JM, Pennober G, Rakotomanana F (2018). Spatial and temporal dynamics of malaria in Madagascar. Malar J..

[7] Vieilledent G, Grinand C, Rakotomalala FA, Ranaivosoa R, Rakotoarijaona J-R, Allnutt TF (2018). Combining global tree cover loss data with historical national forest cover maps to look at six decades of deforestation and forest fragmentation in Madagascar. Biol Conserv..

[8] Harper GJ, Steininger MK, Tucker CJ, Juhn D, Hawkins F (2007). Fifty years of deforestation and forest fragmentation in Madagascar. Environ Conserv..

[9] Scales IR (2011). Farming at the Forest Frontier: Land Use and Landscape Change in Western Madagascar, 1896-2005. Environ Hist Camb..

[10] Schwitzer C, Mittermeier RA, Johnson SE, Donati G, Irwin M, Peacock H (2014). Conservation. Averting lemur extinctions amid Madagascar’s political crisis. Science..

[11] Morelli TL, Smith AB, Mancini AN, Balko EA, Borgerson C, Dolch R (2019). The fate of Madagascar’s rainforest habitat. Nat Clim Chang..

[12] Goodman SM, Benstead JP (2005). Updated estimates of biotic diversity and endemism for Madagascar. Oryx..

[13] Zaehringer JG, Hett C, Ramamonjisoa B, Messerli P (2016). Beyond deforestation monitoring in conservation hotspots: Analysing landscape mosaic dynamics in north-eastern Madagascar. Appl Geogr..

[14] Zohdy S, Derfus K, Headrick EG, Andrianjafy MT, Wright PC, Gillespie TR (2016). Small-scale land-use variability affects Anopheles spp. distribution and concomitant Plasmodium infection in humans and mosquito vectors in southeastern Madagascar. Malar J..

[15] Vittor AY, Gilman RH, Tielsch J, Glass G, Shields T, Lozano WS (2006). The effect of deforestation on the human-biting rate of *Anopheles darlingi*, the primary vector of Falciparum malaria in the Peruvian Amazon. Am J Trop Med Hyg..

[16] Woolhouse ME, Dye C, Etard JF, Smith T, Charlwood JD, Garnett GP (1997). Heterogeneities in the transmission of infectious agents: implications for the design of control programs. Proc Natl Acad Sci U S A..

[17] Stresman GH, Mwesigwa J, Achan J, Giorgi E, Worwui A, Jawara M (2018). Do hotspots fuel malaria transmission: a village-scale spatio-temporal analysis of a 2-year cohort study in The Gambia. BMC Med..

[18] Goodman SM, Benstead JP. The Natural History of Madagascar. University of Chicago Press; 2007.

[19] Dewar RE, Richard AF (2007). Evolution in the hypervariable environment of Madagascar. Proc Natl Acad Sci U S A..

[20] Maps. 2015. https://bioscenemada.cirad.fr/maps/. Accessed 2 Feb 2021.

[21] Moat J, Smith PP, Others. Atlas of the vegetation of Madagascar=. Royal Botanic Gardens, Kew; 2007.

[22] Goodman SM, Raherilalao MJ, Wohlhauser S. Les Aires Protégées Terrestres de Madagascar: Leur Histoire, Description Et Biote. Association Vahatra; 2019.

[23] Myers N, Mittermeier RA, Mittermeier CG, da Fonseca GA, Kent J (2000). Biodiversity hotspots for conservation priorities. Nature..

[24] Seddon N, Tobias J, Yount JW, Ramanampamonjy JR, Butchart S, Randrianizahana H (2000). Conservation issues and priorities in the Mikea Forest of south-west Madagascar. Oryx..

[25] Golden CD, Anjaranirina EJG, Fernald LCH, Hartl DL, Kremen C, Milner DA (2017). Cohort Profile: The Madagascar Health and Environmental Research (MAHERY) study in north-eastern Madagascar. Int J Epidemiol.

[26] Golden CD, Borgerson C, Rice BL, Allen LH, Anjaranirina EJG, Barrett CB (2019). Cohort Description of the Madagascar Health and Environmental Research-Antongil (MAHERY-Antongil) Study in Madagascar. Front Nutr..

[27] Golden CD, Rice BL, Randriamady HJ, Vonona AM, Randrianasolo JF, Tafangy AN (2020). Study Protocol: A Cross-Sectional Examination of Socio-Demographic and Ecological Determinants of Nutrition and Disease Across Madagascar. Front Public Health..

[28] Joncour G (1956). The fight against malaria in Madagascar. Bull World Health Organ..

[29] Smith DL, Guerra CA, Snow RW, Hay SI (2007). Standardizing estimates of the Plasmodium falciparum parasite rate. Malaria J..

[30] Howes RE, Franchard T, Rakotomanga TA, Ramiranirina B, Zikursh M, Cramer EY (2018). Risk Factors for Malaria Infection in Central Madagascar: Insights from a Cross-Sectional Population Survey. Am J Trop Med Hyg..

[31] Rice BL, Golden CD, Anjaranirina EJG, Botelho CM, Volkman SK, Hartl DL (2016). Genetic evidence that the Makira region in northeastern Madagascar is a hotspot of malaria transmission. Malar J..

[32] Ripley BD (2001). The R project in statistical computing. MSOR Connect..

[33] Bejon P, Williams TN, Nyundo C, Hay SI, Benz D, Gething PW (2014). A micro-epidemiological analysis of febrile malaria in Coastal Kenya showing hotspots within hotspots. Elife..

[34] Pull JH, Grab B (1974). A simple epidemiological model for evaluating the malaria inoculation rate and the risk of infection in infants. Bull World Health Organ..

[35] Arambepola R, Keddie SH, Collins EL, Twohig KA, Amratia P, Bertozzi-Villa A (2020). Spatiotemporal mapping of malaria prevalence in Madagascar using routine surveillance and health survey data. Sci Rep..

[36] Magalhães RJ, Langa A, Sousa-Figueiredo JC, Clements AC, Nery SV (2012). Finding malaria hot-spots in northern Angola: the role of individual, household and environmental factors within a meso-endemic area. Malar J..

[37] International Food Policy Research Institute (IFPRI). Global Nutrition Report 2016: From Promise to Impact: Ending Malnutrition by 2030. Intl Food Policy Res Inst; 2016.

[38] Aiga H, Abe K, Andrianome VN, Randriamampionona E, Razafinombana AR, Murai T (2019). Risk factors for malnutrition among school-aged children: a cross-sectional study in rural Madagascar. BMC Public Health..

[39] Rakotomanana H, Gates GE, Hildebrand D, Stoecker BJ. Determinants of stunting in children under 5 years in Madagascar. Matern Child Nutr. 2017;13. 10.1111/mcn.12409. 10.1111/mcn.12409 PMC6865998 28032471

[40] Stevens GA, Finucane MM, De-Regil LM, Paciorek CJ, Flaxman SR, Branca F (2013). Global, regional, and national trends in haemoglobin concentration and prevalence of total and severe anaemia in children and pregnant and non-pregnant women for 1995-2011: a systematic analysis of population-representative data. Lancet Glob Health..

[41] Golden CD, Fernald LCH, Brashares JS, Rasolofoniaina BJR, Kremen C (2011). Benefits of wildlife consumption to child nutrition in a biodiversity hotspot. Proc Natl Acad Sci U S A..

[42] Stewart CP, Fernald LCH, Weber AM, Arnold C, Galasso E (2020). Lipid-Based Nutrient Supplementation Reduces Child Anemia and Increases Micronutrient Status in Madagascar: A Multiarm Cluster-Randomized Controlled Trial. J Nutr..

[43] Bagcchi S (2020). Madagascar’s vulnerable children. Lancet Infect Dis..

[44] United Nations Development Programme (UNDP). Human Development Report 2019: Beyond Income, Beyond Averages, Beyond Today - Inequalities in Human Development in the 21st Century. United Nations; 2019.

[45] Poverty Headcount Ratio at $1.90 a Day (2011 PPP) (% of Population) - Madagascar. World Bank, Development Research Group. https://data.worldbank.org/indicator/SI.POV.DDAY?locations=MG. Accessed 27 Jan 2021

[46] Remonja CR, Rakotoarison R, Rakotonirainy NH, Mangahasimbola RT, Randrianarisoa AB, Jambou R (2017). The importance of public health, poverty reduction programs and women’s empowerment in the reduction of child stunting in rural areas of Moramanga and Morondava, Madagascar. PLoS One..

[47] Marks F, Rabehanta N, Baker S, Panzner U, Park SE, Fobil JN (2016). A Way Forward for Healthcare in Madagascar?. Clin Infect Dis..

[48] Tabor K, Jones KW, Hewson J, Rasolohery A, Rambeloson A, Andrianjohaninarivo T (2017). Evaluating the effectiveness of conservation and development investments in reducing deforestation and fires in Ankeniheny-Zahemena Corridor. Madagascar. PLoS One..

[49] Rice BL, Golden CD, Randriamady HJ, Arisco NJ, Hartl DL (2018). Integrating approaches to study land use change and hotspots of malaria transmission in rural Madagascar: an observational study. Lancet Planet Health..

[50] Howes RE, Mioramalala SA, Ramiranirina B, Franchard T, Rakotorahalahy AJ, Bisanzio D (2016). Contemporary epidemiological overview of malaria in Madagascar: operational utility of reported routine case data for malaria control planning. Malar J..

[51] Nguyen M, Howes RE, Lucas TCD, Battle KE, Cameron E, Gibson HS (2020). Mapping malaria seasonality in Madagascar using health facility data. BMC Med..

[52] Battle KE, Bisanzio D, Gibson HS, Bhatt S, Cameron E, Weiss DJ (2016). Treatment-seeking rates in malaria endemic countries. Malar J..

[53] Bejon P, Williams TN, Liljander A, Noor AM, Wambua J, Ogada E (2010). Stable and unstable malaria hotspots in longitudinal cohort studies in Kenya. PLoS Med..

[54] Stresman G, Bousema T, Cook J (2019). Malaria Hotspots: Is There Epidemiological Evidence for Fine-Scale Spatial Targeting of Interventions?. Trends Parasitol..

