# Supplementary material for: Fine-scale variation in malaria prevalence across ecological regions in Madagascar: a cross-sectional study
Source: BMC Public Health. 2021 May 29;21:1018. doi: 10.1186/s12889-021-11090-3 (PMC8164762; doi:10.1186/s12889-021-11090-3)
Supplement: Supplementary file 1 — Additional file 1: csv contains the malaria rapid diagnostic test outcome data as a line list. Supplementary Table S1.csv contains sampling dates and malaria prevalence data by site and household. Figure S1. shows the distribution of household size (number of individuals per household). Figure S2. shows the difference in prevalence as a function of the length of time between sampling dates All files and the R code used to process the data for this study are also publicly available at the GitHub repository: https://github.com/labmetcalf/madagascar_malaria_prevalence_2021. [file 12889_2021_11090_MOESM1_ESM.zip › Figure S2 20210419.pdf]

Figure S2: Difference in prevalence as a function of the length of time between sampling dates

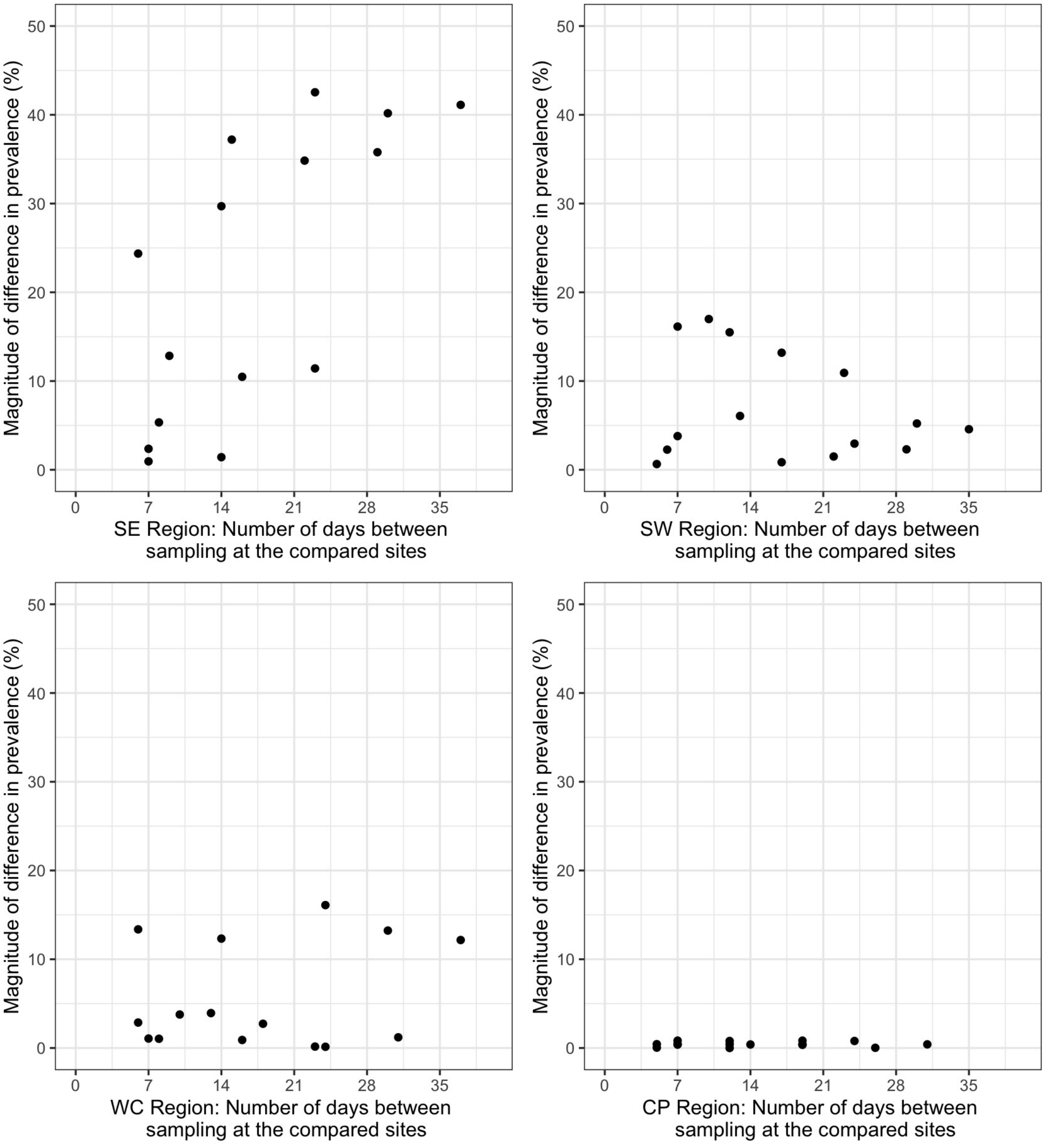

Data not available for the NE region (sampling was performed at all sites in a single month, March 2014 for sites NE.01-NE.02 and April 2017 for sites NE.03-NE.07. Northeast, NE; Southeast, SE; Southwest, SW; West Coast, WC; Central Plateau, CP
